# A structured approach to Shared Decision Making training and assessment of knowledge, attitudes and perception of second year medical students

**DOI:** 10.1080/10872981.2022.2044279

**Published:** 2022-03-09

**Authors:** Charlotte Leblang, Shannon Taylor, April Brown, Jess Knapp, Meenu Jindal

**Affiliations:** a University of South Carolina School of Medicine Greenville, Greenville, SC, USA; b Prisma Health Upstate, Greenville, SC, USA

**Keywords:** Medical education, shared decision making, role-play exercise, curricular integration, SHARE approach

## Abstract

Shared decision making (SDM) has been acknowledged in the last decade. Literature has shown that when physicians are engaged in SDM and form a relationship with their patients, there is higher patient satisfaction of care. Moreover, SDM has been reported to improve patient outcomes and clinical measures. Despite this clear benefit of implementing SDM into clinical practice, there is little evidence for including SDM learning into preclinical medical education. We integrated an exercise for second year medical students to practice the steps of shared decision making. In this paper, the quantitative and qualitative results from a survey of medical students following the SDM learning exercise will be discussed. Students were more educated regarding SDM after this exercise and were motivated to use it in their future clinical careers. They also expressed overall positive attitudes towards SDM tools such as decision aids. Feedback to improve this SDM learning experience included the use of standardized patients, and to expand such education to the clinical environment training. This research provides a model of SDM practice integration into medical education. Similar programs can be beneficial for the development of SDM and other interpersonal skills.

## Introduction

The patient-physician relationship has evolved from a paternal model into a collaborative shared decision-making (SDM) relationship. Shared decision making (SDM) is an approach where clinicians and patients make decisions together based on patients’ preferences and values while utilizing the best available evidence. SDM has been associated with higher patient satisfaction of care and enhanced performance on clinical measures [1,2]. Several barriers to the practice of SDM include the time constraints, physicians’ lack of understanding of the process of SDM and clinical workflow limitations [3].

Integration of SDM courses into undergraduate medical education has been effective in improving medical students’ skills, confidence, and attitudes regarding SDM [3]. Most of such trainings are delivered in the third year of medical school during clerkships and how medical schools deliver and integrate such training into educational curricula is less clear [3,4].

We describe our approach to integrate SDM introduction, training, and assessment in to second year medical students’ education.

Our SDM session included an introductory didactic teaching followed by a role-play exercise and survey questionnaire. The didactic segment focused on AHRQ’s (Agency of Healthcare Research and Quality) SHARE approach and included the slides and videos from the training module 1 [5].

**Figure:**
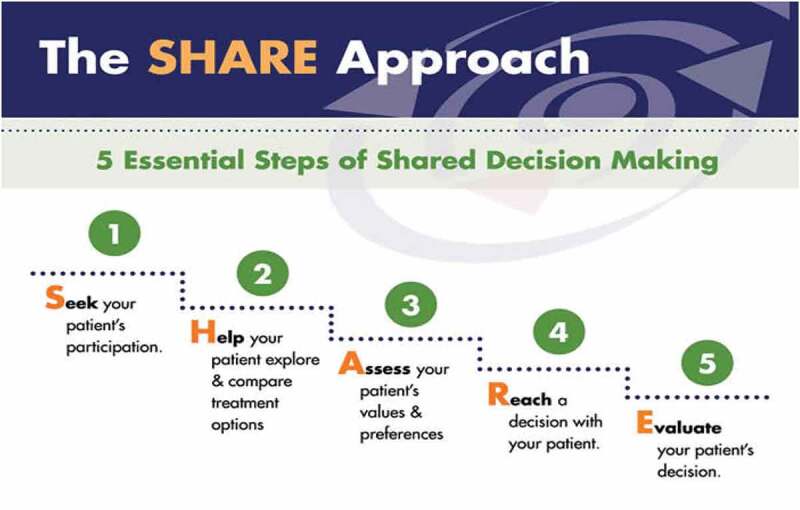
Adapted from:https://www.ahrq.gov/health-literacy/professional-training/shared-decision/index.html [5].

Subsequently, medical students performed role-play exercises in the clinical skills area of the medical school Simulation Center. The role play was conducted amongst the students within their established case-based learning small groups. Patient, physician and observer triads of students participated in the exercise with different clinical scenario scripts (see Appendix 1) and practiced performing SDM. The observer student immediately outside the simulation clinic room graded the physician student utilizing a rubric based on SHARE model (see Appendix 2). The remaining students in the small group and the clinician faculty group leader also observed the interaction live via video, completed the rubric, and discussed the interaction. Then the students switched roles; the students watching remotely performed the SDM skills exercise, while the others watched the live video feed with the faculty member. The scenarios were different in each encounter completed. A total of 103 students participated in the exercise. Twelve faculty small group leaders facilitated the discussion amongst the small group students while observing the exercise and created debrief opportunities. A ten-item post-exercise survey questionnaire assessed students’ knowledge (four questions) and attitudes (four questions) about SDM and their feedback on the content delivery and practice (two questions) with the option to add qualitative comments (see Appendix 3). The survey response rate was 100% as the survey questionnaire was part of the exercise.

96% of the students answered the SDM knowledge questions correctly. 81% agreed or strongly agreed that the session was helpful in adding to their knowledge and practice of shared decision making. Student feedback included consideration of Standardized Patients in future. 96% of the students agreed that performing shared decision making is realistic regardless of time constraints and 84% felt that SDM can be done with patients regardless of their level of education and comfort in discussing medical treatment options. 100% of our student participants agreed that having resources which summarize the risks and benefits of treatment options such as decision aids will be helpful in successful implementation of SDM.

In summary, following SDM training and exercise, our students felt more knowledgeable with increased comfort regarding the practice of SDM and were motivated to use it in their future career. The patient’s education level and time constraints were not of great concern to the students. Feedback to improve this SDM learning experience included the use of Standardized Patients, and to expand such education in the clinical environment. Our students favored a combination of didactics and practical exercise for their SDM education. These findings can inform both undergraduate and graduate medical education curricula as we strive to enhance teaching and experience of our learners in incorporating patient engagement into clinical decision making. As the patient population in the USA becomes increasingly diverse, performing SDM with all patients regardless of their education level or understanding while keeping their preferences and values in mind has the potential to enhance patients’ trust in the health care system, increase uptake of care recommendations and improve clinical outcomes.

This project has several limitations. Didactic component of our training was brief with selective discussion of one of AHRQ’s SDM modules. We did not use a comparison group or pre and post analysis and we did not replicate similar training during clinical years or assess the retention of SDM knowledge and skills.

Future direction of our project includes expansion of similar exercise into clerkship curricula to reinforce the skills and practice. Familiarity and comfort with utilization of decision aids are also of paramount importance. Clinical decision aids have been shown not only to increase patient knowledge, but also to increase the level of shared decision making without lengthening the duration of the encounter [6]. Thus, simulation exercises built around performing SDM while utilizing decision aid would be a great addition to our SDM curriculum. Incorporation of SHARE rubric in to Observed Structured Clinical Exercises will be an important step as well. Voluntary translation of medical documents into plain language by medical students led to higher expression of shared decision-making skills in simulated physician-patient encounters [7]. Such novel ways to enhance learners’ knowledge and comfort in SDM should also be researched. Systematic review of SDM education studies indicated that another important area of future research is the impact of SDM education on students’ behaviors and patient outcomes [8].

In conclusion, a structured approach to enhance medical students’ ability, interest, practice and comfort in taking patients’ perspective by incorporating SDM training and assessment in the medical education curriculum has the promise of promoting patient engagement in health care decisions and quality of care delivered.

## Appendix 1. (Scenarios)

Scenario 1

Doctor

The patient is 11-year-old Katie, who has had multiple bouts of tonsillitis. One treatment option is a tonsillectomy, which may help. However, attacks of tonsillitis get much less frequent as children near Katie’s age. Additionally, there is a risk of severe bleeding after the operation. Your goal is to perform shared decision making with the patient about the treatment approach.

Patient

You are here with your daughter, 11-year-old Katie, because she has had multiple bouts of tonsillitis and is therefore missing school frequently. You are very concerned about this and want Katie to have a tonsillectomy in order to solve the problem. If there are any risk factors associated with surgery, this would cause you to have second thoughts.

Scenario 2

Doctor

The patient has been recently diagnosed with diabetes. His/her current presentation is such that it would be appropriate to treat with lifestyle change or medications. Your goal is to perform shared decision making with the patient about the treatment approach.

Patient

You have been recently diagnosed with diabetes. You are very concerned about this diagnosis because your father suffered many complications of diabetes including neuropathy and amputation. You are here to discuss various options with your physician about further treatment plan. You might share if it comes up that you can’t exercise much due to knee pain. You would prefer a treatment that is most effective at improving blood glucose control to prevent potential future diabetes-related complications.

Scenario 3

Doctor

Patient has been dealing with chronic lower back pain for many years and is currently being managed with opioids (the only type of medication he/she has found to be effective). Surgery is an option, which could reduce the pain, but there is a risk that the pain will not improve or could get worse, and of surgery-related side effects. Your goal is to perform shared decision making with the patient about the treatment approach.

Patient

You have been dealing with chronic lower back pain for many years and it is negatively impacting your life- your ability to work, play with your kids, engage in your hobby of running, etc. You are currently taking opioids (the only type of medication you have found to be effective). You do not want to continue taking opioids due to side effects, dependence, and risk of overdose. Ultimately, you would welcome any other treatment approach if there were a good chance to decrease the pain.

## Appendix 2. (SDM Grading Rubric)

Shared Decision Making Grading Rubric

1.Seek patient’s participation (Invite patient to participate)

Exemplary – Clearly invites patient and reminds patient of the importance of their participation

Satisfactory – Communicates that a decision needs to be made, invites patient to participate

Needs Improvement – Patient role in decision is indirectly implied and patient is not explicitly invited

Does Not Perform

2.Help your patient understand, explore and compare treatment option

(present options with risks and benefits)

Exemplary – Clearly discusses benefits and risks, AND inquiries about patient’s understanding AND compares treatment options

Satisfactory – Clearly discusses benefits or risks AND inquiries about patient’s understanding OR compares treatment options

Needs Improvement – Discusses benefits or risks with no inquiry about patient understanding or comparison of treatment options

Does Not Perform

3.Assess your patient’s values and preferences and assist patient evaluate the options

Exemplary – Assesses/listens to patient’s values and goals actively and assists in evaluating options in alignment with those goals and values.

Satisfactory – Clearly asks about patient values and their goals

Needs Improvement – Minimizes or challenges patient’s values, presents their own goal

Does Not Perform

4.Reach a decision with your patient (facilitate deliberation and decision making)

Exemplary – Helps patient move to a decision. Patient and physician decide together and address next step in implementing decision.

Satisfactory – Helps patient move to a decision. No discussion of next steps.

Needs Improvement – Tells patient what to do without attempt to involve patient in decision OR Asks patient for input but tells the patient what the physician feel would be the best course of action

Does Not Perform

5.Evaluate patient’s comfort and readiness with the decision (assist with implementation)

Exemplary – Inquiries regarding comfort/readiness AND assess any barriers to implementation

Satisfactory – Inquiries regarding comfort/readiness OR assess barriers to implementation

Needs Improvement – not clear assessment of patient’s readiness or comfort

Does Not Perform

## Appendix 3. (Questionnaire)

Survey Questionnaire:

1.Which of the following statements is true in the process of shared decision making:

- Patient has the primary responsibility of making a decision.
- Each participant (patient and physician) shares responsibility in the decision about how to proceed.
- Both parties (patient and physician) share information, but clinician makes the decision for the patient.
- Patient usually doesn’t express his or her preferences and values especially if it contradicts with those of physicians’.

2.A young woman develops radiating pain as a result of a back injury. Her medical exam and magnetic resonance imaging reveal a lumbar disk protrusion. Which of the following is a part of shared decision making:

- Her physician strongly believes in surgical option and tries to convince them to go for surgery.
- Physician describes different approaches that include surgery, nerve blocks, a back brace, physical therapy, and watchful waiting.
- The patient asks about risks of surgery, the physician is very busy and tells the patient to really not worry about it.
- The patient and her parents are averse to surgery but don’t want to share their concerns with the physician.
  3.A young woman develops a breast lump and is diagnosed with uncomplicated early breast cancer. Due to the early stage of illness, the physician clearly believes that the patient is an excellent candidate for lumpectomy. Because of a strong family history, however, the young woman prefers bilateral mastectomy. Which of the following in further management describes patient centered care and shared decision making ?

- After further discussion with the patient and her husband, the physician understands and accepts the patient’s decision and performs the more radical surgery.
- The physician feels very uncomfortable with patient’s decision and would rather pursue lumpectomy.
- Patient threatens to take legal action against the physician if he doesn’t follow their wishes.
- Patient’s husband would prefer lumpectomy too and he tries to convince his wife to avoid bilateral mastectomy.

4.Which of the following is an example of decision aid that can be used to assist with shared decision making for prostate cancer screening:

- A video in the waiting room discussing risks and benefits of PSA testing.
- A pamphlet that patient can take home and review between appointments while trying to make the decision for PSA testing.
- A computer based algorithm designed for patients to assist with the decision for PSA testing.
- All of the above.

5.Shared decision making can only be done with patients who are sufficiently educated and confident to discuss treatment or screening options with their clinician.

- Strongly agree
- Agree
- Disagree
- Strongly disagree

6.Doing shared decision making is unrealistic because it takes too much time.
  - Strongly agree
  - Agree
  - Disagree
  - Strongly disagree
7.Having resources which summarize the risks and benefits of clinical decisions would be

helpful (e.g., a patient decision aid).

- Strongly agree
- Agree
- Disagree
- Strongly disagree

8.Even if the patient does not wish to be involved in the decision-making process, it is the clinician’s role to encourage the patient to make a decision.
  - Strongly agree
  - Agree
  - Disagree
  - Strongly disagree
9.Following form of learning about shared decision making I have found/ or think I will find helpful.
a. Didactic learning
b. Practical shared decision-making training (using role plays and or simulated patients).
c. Combination of both
d. None of these

10.I found today’s session helpful in adding to my knowledge and practice of shared decision making.

- Strongly agree
- Agree
- Disagree
- Strongly disagree
